# Decomposing heritability and genetic covariance by direct and indirect effect paths

**DOI:** 10.1371/journal.pgen.1010620

**Published:** 2023-01-23

**Authors:** Jie Song, Yiqing Zou, Yuchang Wu, Jiacheng Miao, Ze Yu, Jason M. Fletcher, Qiongshi Lu

**Affiliations:** 1 Department of Statistics, University of Wisconsin-Madison, Madison, Wisconsin, United States of America; 2 Department of Statistics, Stanford University, Stanford, CA, United States of America; 3 Department of Biostatistics and Medical Informatics, University of Wisconsin–Madison, Wisconsin, United States of America; 4 Center for Demography of Health and Aging, University of Wisconsin-Madison, Madison, Wisconsin, United States of America; 5 Department of Sociology, University of Wisconsin-Madison, Madison, Wisconsin, United States of America; 6 La Follette School of Public Affairs, University of Wisconsin-Madison, Madison, Wisconsin, United States of America; Newcastle University, UNITED KINGDOM

## Abstract

Estimation of heritability and genetic covariance is crucial for quantifying and understanding complex trait genetic architecture and is employed in almost all recent genome-wide association studies (GWAS). However, many existing approaches for heritability estimation and almost all methods for estimating genetic correlation ignore the presence of indirect genetic effects, i.e., genotype-phenotype associations confounded by the parental genome and family environment, and may thus lead to incorrect interpretation especially for human sociobehavioral phenotypes. In this work, we introduce a statistical framework to decompose heritability and genetic covariance into multiple components representing direct and indirect effect paths. Applied to five traits in UK Biobank, we found substantial involvement of indirect genetic components in shared genetic architecture across traits. These results demonstrate the effectiveness of our approach and highlight the importance of accounting for indirect effects in variance component analysis of complex traits.

## Author summary

Complex human traits are influenced by both genetic and environmental factors. Recent evidence suggests that genetic effects on human traits can sometimes be mediated by the environment. One example is that parental genomes could influence parental behavior and family environment, which, in turn, shape the phenotypes of their children. Since parent and offspring genotypes are correlated, these environmentally mediated effects will be captured by conventional genome-wide association studies and may potentially bias our interpretation of genotype-phenotype associations. In this work, we propose a principled statistical framework named PARSEC to partition the genetic components of single traits and the shared genetic components between traits into direct and indirect effect paths. It marks an important methodological innovation by resolving a limitation in almost all existing genetic correlation estimation approaches and may have broad and impactful applications in future GWAS analysis for complex traits that are affected by parental genetics and family environment.

## Introduction

Genetic correlation is an effective metric for quantifying the shared genetic architecture of multiple complex traits [1,2] and has quickly gained popularity in genome-wide association studies (GWAS) in the past few years. Genetic correlation is typically defined as the correlation between additive genetic components of two complex traits and can be estimated from genome-wide data of millions of single nucleotide polymorphisms (SNPs). Several methods have been developed to improve genetic correlation estimation using individual-level GWAS data [3,4], GWAS summary statistics [5,6], or both [7]. Recent studies have also expanded this concept to quantify genetic correlations in local genomic regions [8–11], between human ancestral populations [12,13], and using other types of genetic variations [14]. Overall, these methods have become a routine component of complex trait genetic studies and provided insights into the genetic basis of numerous human traits.

As genetic correlation analysis becomes standard in GWAS, challenges arise in interpreting the pervasive correlations observed across many phenotypes. We now know that parents’ genomes can influence parental behaviors and family environment which, in turn, shape the phenotypes of their children. Additionally, because parent and offspring genotypes are correlated, typical GWAS can be severely confounded by parental genotypes, and effect estimates obtained in GWAS are mixtures of direct genetic effects (i.e., how one’s own genotypes affect his/her phenotype) and indirect genetic effects (i.e., how parents’ genotypes affect their children’s phenotypes; this is also referred to as genetic nurture) [15,16]. Therefore, interpretation of genetic correlations involving human behavioral phenotypes is particularly challenging because most methods for estimating genetic correlations [6,17] ignore these apparent gene-environment correlations underlying human behavior [18–25]. Being able to decompose shared genetics between two traits by direct and indirect effect paths is crucial for understanding the respective roles of genetics and environments. Although methods have been developed for separating SNP effects into direct/indirect paths, and follow-up genetic correlation analyses performed on direct/indirect association statistics can be a feasible strategy in some cases [26–29], these GWAS typically require data from a large number of families which can be difficult to obtain even in large biobanks.

In the past few years, an extensive literature has investigated the decomposition of phenotypic variance based on gene-environment relations. Methods like M-GCTA [30,31], Trio-GCTA [32], RDR [33], and GREML-KIN [34] have leveraged family structures in genetic samples to improve heritability estimation and quantify variance components for maternal, paternal, offspring, and sibling genetic effects. Several methods have also allowed imputation of parental genotypes which enables estimation of parental and offspring genetic effects in downstream GWAS applications [25,35,36]. These methods decompose the phenotypic variance of a single complex trait into multiple variance components according to direct and indirect effect paths, but none of them can be applied to partition genetic covariance–the shared additive genetic component between a pair of complex traits.

In this paper, we introduce a statistical framework named PARSEC (PARental Stratification of genetic Effect Components) to estimate the variance and covariance components of direct/indirect genetic effects on trait pairs using data from a limited number of families. This framework enables us to partition both heritability and genetic covariance into direct and indirect pathways, and gain knowledge of how genetics and environment jointly contribute to complex traits as well as the correlation between trait pairs. We employ the method of moments to obtain accurate estimates of variance and covariance parameters from family-based data and use a Jackknife approach to obtain confidence intervals. We apply our framework to five complex traits in UK Biobank (UKB) [37] and partition the trait-specific and shared genetic components by direct and indirect effect paths.

## Results

### Method overview

Our approach is built on a pair of linear models for a pair of standardized traits Y_1_ and Y_2_, standardized own- genotypes X, and standardized parental genotypes X_p_+X_m_:

$$Y_{1}=X\alpha_{1}+(X_{p}+X_{m})\beta_{1}+\epsilon_{1}$$

$$Y_{2}=X\alpha_{2}+(X_{p}+X_{m})\beta_{2}+\epsilon_{2}$$

Error terms ϵ_1_ and ϵ_2_ are assumed to have mean 0 and variance-covariance matrix Σ. α_k_ and β_k_ (k = 1, 2) are random effect vectors denoting direct (of own- genotypes) and indirect effects (of parental genotypes) on two traits. They share the variance-covariance structure below with M being the number of SNPs and I_M_ being the identity matrix.


$$\mathrm{var}[\begin{array}{c} \alpha_{1}\\ \alpha_{2}\\ \beta_{1}\\ \beta_{2} \end{array}]=\frac{1}{M}[\begin{array}{cccc} \sigma_{\alpha_{1}}^{2}I_{M} & \rho_{\alpha }I_{M} & \rho_{\alpha_{1}\beta_{1}}I_{M} & \rho_{\alpha_{1}\beta_{2}}I_{M}\\ \rho_{\alpha }I_{M} & \sigma_{\alpha_{2}}^{2}I_{M} & \rho_{\alpha_{2}\beta_{1}}I_{M} & \rho_{\alpha_{2}\beta_{2}}I_{M}\\ \rho_{\alpha_{1}\beta_{1}}I_{M} & \rho_{\alpha_{2}\beta_{1}}I_{M} & \sigma_{\beta_{1}}^{2}I_{M} & \rho_{\beta }I_{M}\\ \rho_{\alpha_{1}\beta_{2}}I_{M} & \rho_{\alpha_{2}\beta_{2}}I_{M} & \rho_{\beta }I_{M} & \sigma_{\beta_{2}}^{2}I_{M} \end{array}]$$

Here, ρ_α_ and ρ_β_ are two key parameters we focus on, although other cross terms are also estimated and may be of interest in practice. These two parameters quantify the covariance of direct effects and covariance of indirect effects on two traits, respectively. We apply the method of moments to produce parameter estimates and employ a Jackknife approach to quantify the standard error of parameter estimates. More statistical details are described in the Methods section.

### Simulation results

**Fig 1:**
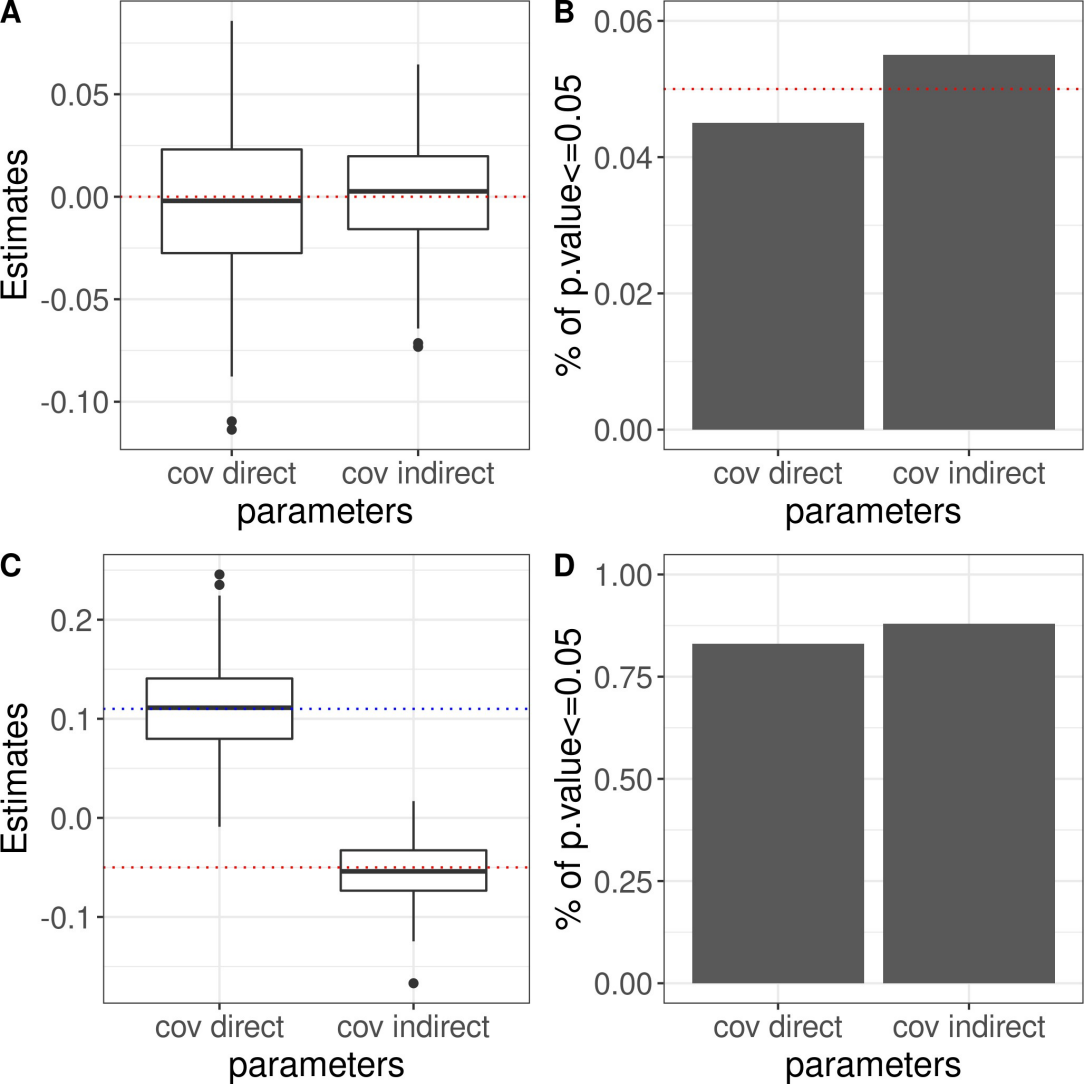
Simulation results on point estimates, type-I error, and statistical power for covariance of direct/indirect effects under different simulation settings. The first row and the second row are respect to two simulation settings respectively. Panels A and C: box plots for point estimates of *ρ*_*α*_ (i.e., covariance of direct effects) and *ρ*_*β*_ (i.e., covariance of indirect effects). Red dotted lines indicate the true values of *ρ*_*α*_. Blue dotted lines show the true value of *ρ*_*β*_. Panels B and D: proportion of p-values < = 0.05 across 200 repeats. In panel B, the dotted line highlights the type-I error threshold 0.05.

We first performed simulations to demonstrate that our method produces unbiased estimates with well-calibrated confidence intervals. We randomly selected 2000 families from UKB with data on full sibling pairs and imputed parental genotypes to perform simulations. The imputation is based on expectation of the average of parental genotypes using two or more sibling genotypes [36]. We explored multiple parameter settings. Each setting contained 200 repeats. Details on simulation settings are described in the Methods section and in **S1 Table**. Results related to covariance of direct effects and covariance of indirect effects are shown in **Fig 1**. Results for the estimates of all 14 parameters are shown in **S1 Fig.** We observed unbiased estimates and well-controlled confidence interval coverage rates for all parameters across various settings.

### Partitioning heritability by direct/indirect effect paths for five complex traits

We applied our model to 12,571 families in UKB with full sibling data available and parental genotypes imputed (Methods). A focal trait we studied in this paper is educational attainment due to its known substantial component in the indirect genetic effect path [15]. In our previous work [26], we partitioned direct and indirect genetic components for educational attainment and estimated their genetic correlations with many traits. Notable findings from that study include correlations with anthropometric traits (e.g. height) as well as numerous health outcomes. In addition, income GWAS is known to have a very high genetic correlation with education [38]. Thus, we chose educational attainment (EA) with height, body mass index (BMI), overall health, and income as the phenotypes of interest in this study. For each trait pair, we estimated 14 parameters including trait-specific parameters (which partition heritability), trait pair parameters (which partition genetic covariance), and parameters for error terms. The detailed data processing procedure is described in the Methods section.

**Fig 2:**
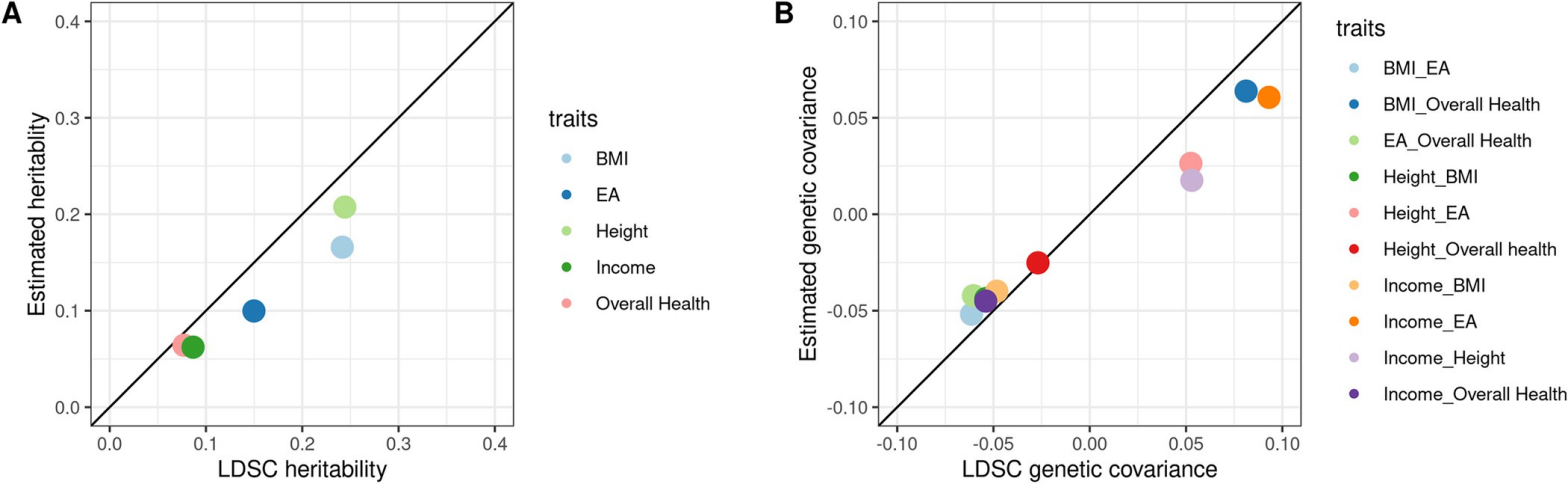
Total heritability and genetic covariance can be recovered from partitioned parameter estimates. A: estimates for total heritability. B: estimates for genetic covariance. X-axis: LDSC estimates. Y-axis: Estimates reconstructed from PARSEC estimates in our analysis.

First, we examined parameter estimates for the variance of direct genetic effects, variance of indirect genetic effects, and covariance between direct and indirect effects on the same trait. These parameters are the partitioned terms of trait heritability; therefore, we can reconstruct total heritability estimates by linearly combining these three terms. For comparison, we also calculated LDSC heritability estimates using GWAS performed on the same data. We observed a high correlation (correlation = 0.962; **Fig 2A**).

**Fig 3:**
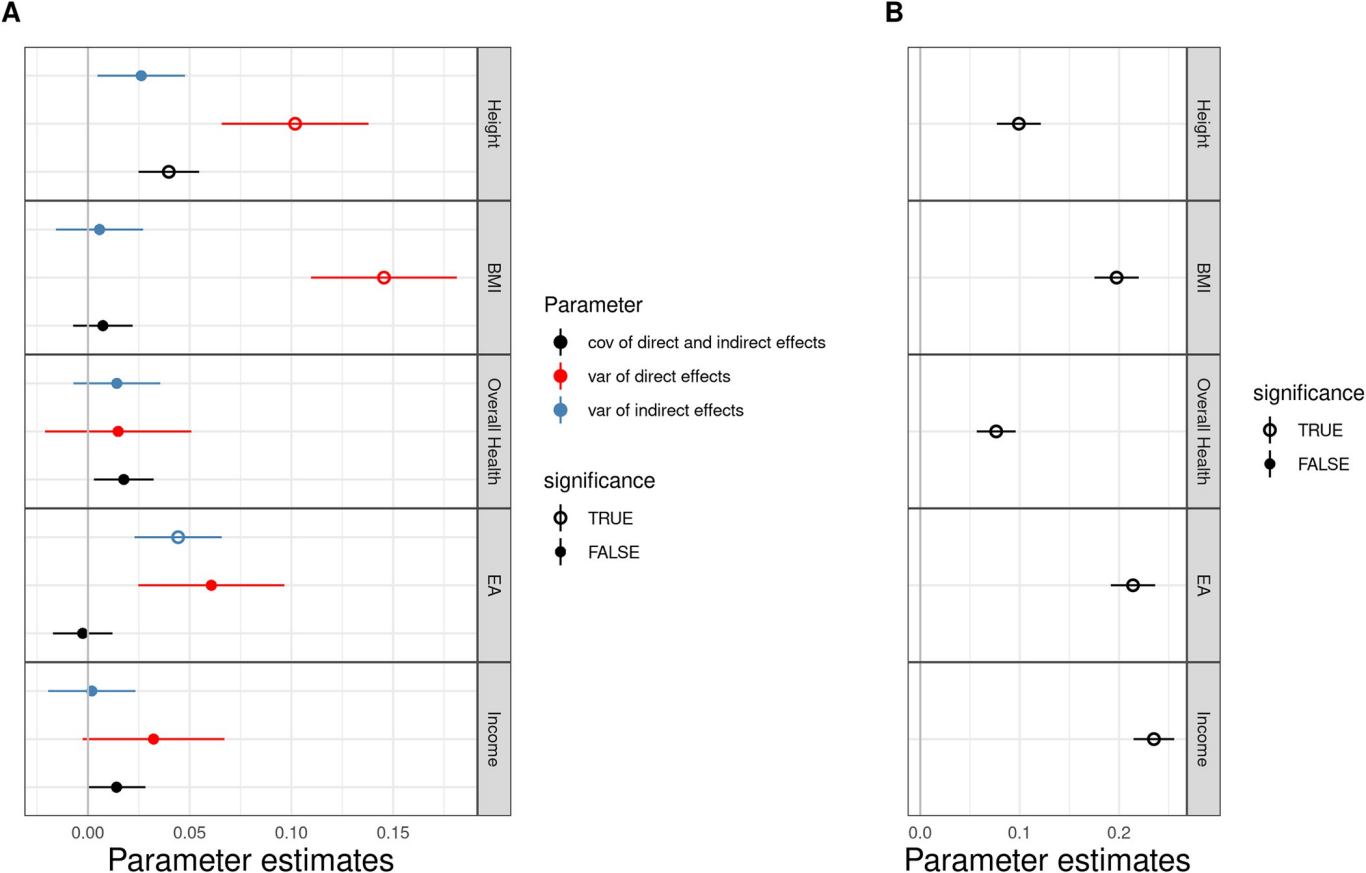
Estimation results for 5 complex traits in UKB. Each interval shows the point estimate and standard error for one parameter. Circles denote significant parameter estimates at the 0.05 level. Other estimates are denoted by solid circles. A: variance of direct effects, variance of indirect effects, and covariance of direct and indirect effects for height, BMI, overall health, EA and income. B: covariance of error terms for sibling pairs for each trait.

Results of these three parameters are shown in **Fig 3A** and **S2 Table**. We found a substantial and statistically significant direct effect component on BMI (p = 4.9×10^−5^). The BMI indirect effect was close to zero and not significant. Similarly, we found a significant direct genetic component (p = 4.8×10^−3^) and a weaker indirect component for height. Notably, the direct and indirect genetic components for height showed a significant positive covariance (p = 7.7×10^−3^). The direct and indirect genetic components for EA were similar in size but only the indirect component reached statistical significance. Consistent with multiple previous studies [26,36], we did not find evidence for a genetic correlation between the EA direct and indirect effects, although the reason for the lack of correlation remains unclear. We also found null results for household income and overall health.

Some other parameters in PARSEC also have important implications. For example, we obtained the estimated covariance between siblings’ error terms for each trait which quantifies the degree to which siblings’ phenotypic correlation is explained by their shared environment (**Fig 3B** and **S2 Table**). Error term covariances are significant for all 5 traits in our analysis. Branigan et al. [39] previously measured the contribution of shared environment between twins on EA using ACE model. They obtained an estimate of around 0.3 from two UK cohorts. However, this strategy does not distinguish environmental contributions from the factors that can be explained by parental genetics. PARSEC provides a useful alternative strategy to estimate the environmental contribution between full siblings and tease part the contribution of parental genomes. We obtained an estimate of 0.22 for EA.

### Partitioning genetic covariance among five complex traits

PARSEC also partitions the total genetic covariance between two traits into direct and indirect effect paths. Similar to the analysis for total heritability, we reconstructed the total genetic covariance estimates based on partitioned parameters (Methods) and compared them to LDSC estimates. We found a high correlation between these estimates (**Fig 2B**, correlation = 0.977).

**Fig 4:**
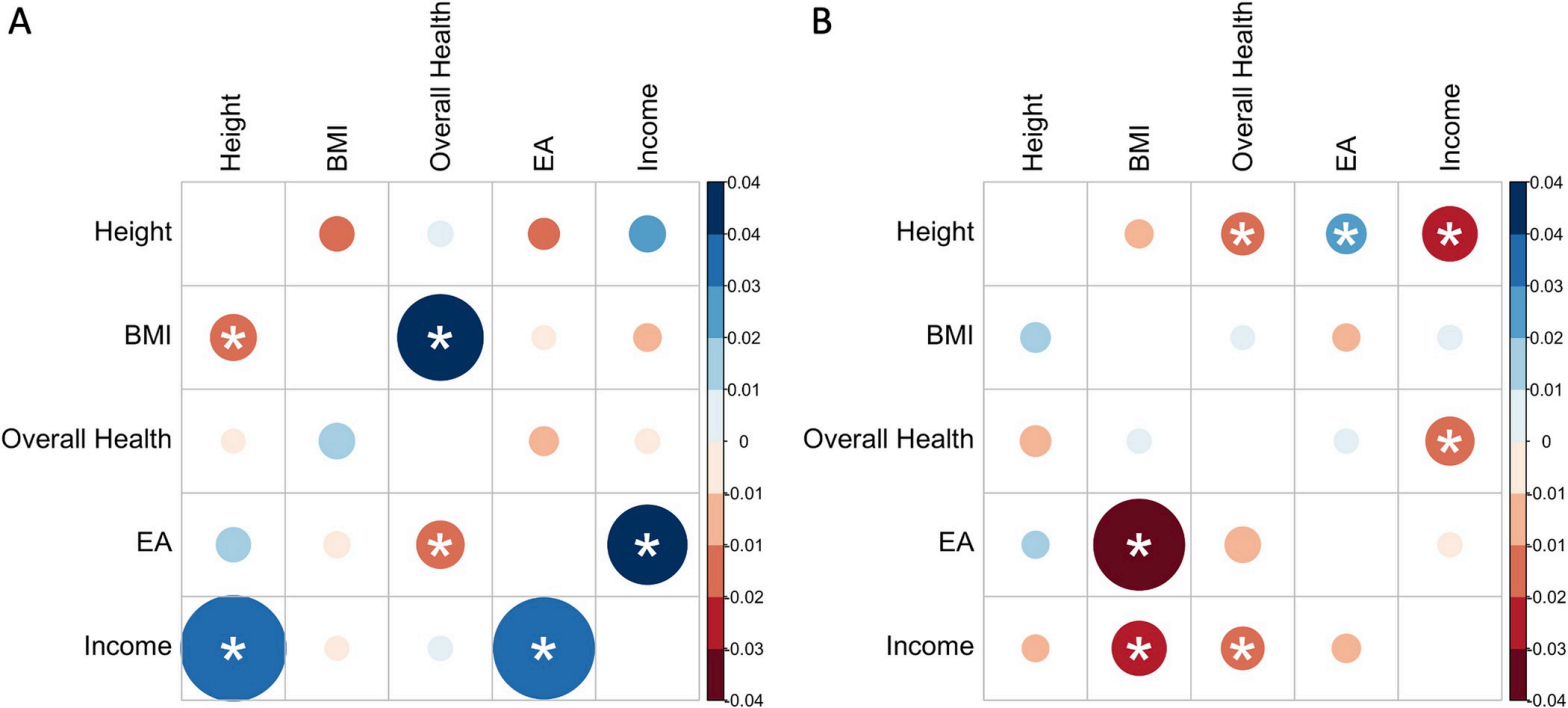
Estimation results for covariance of direct/indirect effects on five complex traits. A: upper triangle: heatmap of direct effect genetic covariance (i.e., *ρ*_*α*_); lower triangle: heatmap of indirect effect genetic covariance (i.e., *ρ*_*β*_). B: covariance between direct effects of trait 1 (rows) and indirect effects of trait 2 (columns). Size of the circles visualizes false discovery rate (FDR). Smaller size refers to larger FDR. Significant correlations with FDR<0.05 are marked by asterisks.

Estimation results for *ρ*_*α*_ (i.e., genetic covariance of direct effects on two traits) and *ρ*_*β*_ (i.e., genetic covariance of indirect effects on two traits) are shown in **Fig 4A and 4B** (also see **S3 Table**). We found a significant direct effect covariance (*ρ*_*α*_ = 0.044) between BMI and worse overall health (a higher value in this trait indicates worse health; see Methods). This contributes to 69% of the total genetic covariance between BMI and overall health. Several other trait pairs showed significant covariances of indirect genetic components, including height and BMI (*ρ*_*β*_ = -0.02), height and income (*ρ*_*β*_ = 0.032), and EA and overall health (*ρ*_*β*_ = -0.018). EA and income have substantial and statistically significant covariances in both direct and indirect genetic components (*ρ*_*α*_ = 0.044 and *ρ*_*β*_ = 0.032). Across all trait pairs, direct effects explain an average of 79.6% of total genetic covariance, suggesting substantial contribution of indirect effect components and their correlation with direct effects.

We also estimated other cross-term covariances, including the covariance of direct effect on one trait and indirect effect on another trait (**Fig 4C and 4D**). We identified a number of significant correlations. For example, we found highly significant correlations between the indirect effect of BMI and the direct effect of both EA ($\rho_{\beta_{1}\alpha_{2}}$ = -0.037) and income ($\rho_{\beta_{1}\alpha_{2}}$ = -0.028). The direct effect of income is also negatively correlated with the indirect effects of overall health ($\rho_{\beta_{1}\alpha_{2}}$ = -0.02). The direct effect of height is correlated with the indirect effect of better overall health ($\rho_{\alpha_{1}\beta_{2}}$ = -0.02), higher EA ($\rho_{\alpha_{1}\beta_{2}}$ = 0.02), and lower income ($\rho_{\alpha_{1}\beta_{2}}$ = - 0.029).

## Discussion

Genetic correlation/covariance analysis has rapidly gained popularity in the past few years. Almost all GWAS papers these days estimate genetic correlation to gain insights into the shared genetics across multiple complex traits. However, interpretation of these correlations can be unsatisfying. Recent efforts in dissecting the direct and indirect trait genetic components have advanced this topic. For example, Warrington et al. estimated the direct and indirect (maternal) effects on birth weight, then correlated them with other complex traits [40]. They concluded that the genetic correlation between birth weight and higher educational attainment is mostly explained by the maternal effect on birthweight, not the direct effect. That is, mothers with higher genetic propensity for EA tend to have children with higher birth weight, while the fetus’ genetic propensity on education and birth weight seem uncorrelated. Similarly, in our recent study, we estimated direct and indirect genetic effects on EA leveraging multi-generational GWAS data [26]. We found that the well-documented genetic correlation between lower EA and higher depression risk is mostly explained by the indirect component of EA. That is, parents who have depression risk may negatively impact children’s school performance. Although inference of causality remains challenging, partitioning shared genetic components into direct and indirect paths has led to some incredibly interesting hypotheses that were previously neglected in the literature.

In these examples, people were able to partition the direct/indirect effects of one trait, and then quantify how they correlate with the combined effects of a second trait. In this paper, we introduced PARSEC, a new statistical framework to decompose heritability and genetic covariance of complex traits by direct and indirect effect paths. Our approach provides a more refined partition of the shared genetic components, allowing both traits’ direct/indirect effects to be investigated which provides a more complete picture of the complex genetic sharing underlying multiple phenotypes. Through simulations and application to five traits using full siblings and their imputed parental genotypes in UKB, we demonstrated that our model produces unbiased estimates with well-controlled confidence interval coverage.

Partitioned variance and covariance components can be combined to recover traditional heritability and genetic covariance estimates. But stratified heritability and genetic covariance estimates provided important new insights into the genetic basis of complex traits. First, our analysis confirmed that traits like EA have a substantial indirect genetic component. One interpretation is that a substantial proportion of trait heritability is mediated through parents and the family environment they create although other mechanisms remain possible [41]. For some other traits (e.g., BMI), the contribution of indirect genetic effect is negligible, and heritability is primarily explained by the direct genetic component. More importantly, our approach allowed decomposing genetic covariance, which is novel compared to other approaches in the literature. Similar to findings in single-trait analysis, our results highlight the importance of considering indirect genetic components when estimating and interpreting the shared genetic basis between complex traits. Several trait pairs in our analysis (e.g., height and income) only showed significant genetic covariance in their indirect genetic components. Some other traits (e.g., BMI and overall health) have substantial genetic sharing of the direct effect component. For EA and income, genetic covariances of both direct and indirect genetic components were substantial and highly statistically significant. Omitting indirect effect components in genetic correlation analysis fails to identify the family environment as a key factor contributing to cross-trait genetic correlation and will likely lead to biased interpretation. With our approach, it is now possible to carefully decompose heritability and shared genetic components into direct and family-mediated paths, which will benefit post-GWAS analysis of human socio-behavioral phenotypes.

Our study also has several limitations. First, parental genotypes used in our analysis were not directly measured but imputed based on siblings’ genotypes. The imputation accuracy depends on several factors including the degree of assortative mating in the population and number of siblings in each family. These may affect the accuracy of parameter estimates in PARSEC. But based on analysis that was conducted by SNIPar, the estimates for multiple regression were proved to remain unbiased and consistent. On the other hand, while our approach can be applied to measured genotypes and phenotypes in parent-offspring trios, available data in existing population cohorts often have smaller sample sizes which may lead to reduced statistical power. Second, we focused on genetic covariance instead of genetic correlation in our cross-trait analysis. Although it is also possible to calculate genetic correlations by combining stratified genetic covariance and heritability estimates, these correlation estimates tend to be numerically unstable at the sample size in our analysis. Third, our analyses were limited to quantitative traits. In our current analysis, multiple traits (i.e., EA, income, and overall health) were converted into continuous values from categorical input. For applications involving binary or categorical outcomes, future work is needed to expand the statistical model. Fourth, our analysis does not distinguish maternal and paternal indirect effects due to constraints in the data (i.e., only average parental genotype can be imputed), although we note that our model can be easily extended to estimate maternal and paternal effects separately if genotype data are available for both parents. We also assumed absence of sibling effects and indirect genetic effects from other distant family members. This assumption is partly supported by evidence in the literature [36], but these effects may be of interest for specific traits. An interesting future direction is to include sibling effects using cohorts with larger samples. Fifth, methods have been developed to leverage multi-generational GWAS summary statistics to quantify direct and indirect genetic effects at the SNP level [26]. While heritability and genetic covariance estimation based on individual-level data is known to be statistically more efficient compared to estimates produced from GWAS summary statistics [6,42], summary statistics-based analysis has the advantage of combining data from many studies while a single cohort of individual-level information is unavailable. Future studies need to quantitatively compare different analytical strategies in realistic data settings in order to understand the pros and cons of each approach and guide future studies. Finally, we assumed random mating and a purely additive genetic architecture in our model. The influence of assortative mating on heritability and genetic correlation estimation has been convincingly demonstrated in recent studies [43,44]. Genome-wide dominant effects [45] and epistasis [46] have also been studied for various complex traits with mixed results. It is an important future direction to expand the PARSEC model and develop estimators robust to these issues.

Taken together, our method is a first step to explore the covariance structure of direct and indirect effects for complex human traits. It marks an important methodological innovation and may have broad and impactful applications in future GWAS analysis for traits that are affected by parental genetics and family environment.

## Methods

### PARSEC model details

We assume a pair of linear genetic models as follows:

$$Y_{1}=X\alpha_{1}+(X_{p}+X_{m})\beta_{1}+\epsilon_{1}$$

$$Y_{2}=X\alpha_{2}+(X_{p}+X_{m})\beta_{2}+\epsilon_{2}$$

where *Y*_1_ and *Y*_2_ denote two complex traits, *X* denotes own genotypes, and *X*_*p*_ and *X*_*m*_ denote paternal and maternal genotypes. Since we used sibling pairs data as input (that will be described later in this section), we assume the total number of sibling pairs is N. Then *Y*_1_ and *Y*_2_ are vectors with length 2N, and *X*, *X*_*p*_ and *X*_*m*_ are 2N-by-M matrices, where M is the total number of SNPs. We assume both genotypes and phenotypes (*Y*_1_, *Y*_2_, *X*, *X*_*p*_, and *X*_*m*_) to be standardized. *α*_1_ and *α*_2_ are random effect terms that quantify the direct genetic effects on *Y*_1_ and *Y*_2_, i.e., how someone’s own genotypes affect their own phenotype. Following conventions in the linear mixed model literature on modeling heritability [47], we also assume these effects to follow normal distributions.


$$\alpha_{k}∼N\left(0,\frac{\sigma_{\alpha_{k}}^{2}}{M}I_{M}\right),\;k=1,2$$

Here, $\sigma_{\alpha_{1}}^{2}$ and $\sigma_{\alpha_{2}}^{2}$ are the direct genetic variance components for two traits respectively. Similarly, *β*_1_ and *β*_2_ are random effect terms for quantifying indirect genetic effects on two traits, i.e., how the parents’ genotypes affect someone’s phenotype. These random effects also follow normal distributions.


$$\beta_{k}∼N\left(0,\frac{\sigma_{\beta_{k}}^{2}}{M}I_{M}\right),\;k=1,2$$

Importantly, direct and indirect effects on the same trait (i.e., *α*_*k*_ and *β*_*k*_) can be correlated. Direct effects on two different traits (i.e., *α*_1_ and *α*_2_), or indirect effects on two traits (i.e., *β*_1_ and *β*_2_), can both be correlated. Fairly generally, we assume *α*_1_, *α*_2_, *β*_1_, and *β*_2_ to have a joint distribution:

$$\left[\begin{array}{c} \alpha_{1}\\ \alpha_{2}\\ \beta_{1}\\ \beta_{2} \end{array}\right]∼N(0,\frac{1}{M}\left[\begin{array}{cccc} \sigma_{\alpha_{1}}^{2}I_{M} & \rho_{\alpha }I_{M} & \rho_{\alpha_{1}\beta_{1}}I_{M} & \rho_{\alpha_{1}\beta_{2}}I_{M}\\ \rho_{\alpha }I_{M} & \sigma_{\alpha_{2}}^{2}I_{M} & \rho_{\alpha_{2}\beta_{1}}I_{M} & \rho_{\alpha_{2}\beta_{2}}I_{M}\\ \rho_{\alpha_{1}\beta_{1}}I_{M} & \rho_{\alpha_{2}\beta_{1}}I_{M} & \sigma_{\beta_{1}}^{2}I_{M} & \rho_{\beta }I_{M}\\ \rho_{\alpha_{1}\beta_{2}}I_{M} & \rho_{\alpha_{2}\beta_{2}}I_{M} & \rho_{\beta }I_{M} & \sigma_{\beta_{2}}^{2}I_{M} \end{array}\right])$$

Here, *ρ*_*α*_ quantifies the covariance of direct effects on two traits and *ρ*_*β*_ is the covariance of indirect effects on two traits. Similarly, $\rho_{\alpha_{1}\beta_{1}},\rho_{\alpha_{1}\beta_{2}},\rho_{\alpha_{2}\beta_{1}}$, and $\rho_{\alpha_{2}\beta_{2}}$ are pairwise covariance parameters between *α* and *β*. We note that this model is similar to what is used in the genetic nurture literature [15,26] in that it quantifies how parental genotypes shapes children’s phenotypes. But a difference is that it is a polygenic model that uses random variables *α*_*k*_ and *β*_*k*_ to characterize genome-wide effects. This is motivated by the linear mixed model literature on heritability and genetic covariance estimation.

Based on the model formulation, naturally one would assume that the data required to fit this model would be genotypes of parent-offspring trios (i.e., *X*, *X*_*p*_, and *X*_*m*_) and offspring phenotypes on two traits (i.e., *Y*_1_ and *Y*_2_). While this is true, the number of trios available even in large population biobanks such as UKB is limited. Therefore, we made two important adjustments. One is that in our analyses, we leveraged the relatively large number of full sibling pairs available in UKB and recent statistical genetic advances in parental genotype imputation [36]. We begin with N sibling pairs (thus the total sample size is 2N), then impute the sum of parental genotypes *X*_*p*_+*X*_*m*_. These become the input data in our analysis. This also leads to the second adjustment we made which has also been shown above–we do not distinguish paternal and maternal indirect effects and instead focus on their average. This is similar to what was done in the indirect effect GWAS literature when sample size was small [26]. In practice, in cases where a large number of trios are available, paternal and maternal indirect effects may be denoted as separate random variables in this framework. As we mentioned, the indirect effect component quantifies the averaged maternal and paternal effects. If one dominates the other (such as maternal indirect effects dominates paternal effects), the estimate still has a clear interpretation. We illustrate this in more details as below:

Suppose the true model for children’s phenotype *Y* in a family is

$$Y=X\alpha +X_{p}\beta_{p}+X_{m}\beta_{m}+\epsilon$$

Where *α*, *β*_*p*_, *and β*_*m*_ are the direct, indirect paternal, and indirect maternal effects, respectively, and *ϵ* denotes the noise term. Without loss of generality, we assume *Y*, *X*, *X*_*p*_, and *X*_*m*_ are standardized to have mean 0 and variance 1. If we further denote transmitted alleles and non-transmitted alleles as *T* and *NT* respectively, then the model above can be rewritten as:

$$Y=X\alpha +(T_{p}+NT_{p})\beta_{p}+(T_{m}+NT_{m})\beta_{m}+\epsilon$$

We also note that the child’s genotype is the sum of maternally and paternally transmitted alleles, i.e., *X* = *T*_*p*_+*T*_*m*_.

Now, recall that the actual model we fit in the PARSEC approach is

$$Y=X\alpha +(X_{p}+X_{m})\beta +\epsilon$$

Here, maternal and paternal genotypes are combined and *β* quantifies the indirect genetic component in this framework. Fitting this model on data generated from the true model we introduced above will give us a *β* estimate that is the projection of *T*_*p*_*β*_*p*_+*T*_*m*_*β*_*m*_ on *X*_*p*_+*X*_*m*_. It can be further shown that this projection is:

$$\beta =\frac{\beta_{p}+\beta_{m}}{2}$$

We have previously derived this relationship in an earlier paper [26]. Therefore, the estimated indirect effect *β* is the average of indirect maternal genetic effect *β*_*m*_ and indirect paternal genetic effect *β*_*p*_, which also means $var(\beta )=var(\frac{\beta_{p}+\beta_{m}}{2})$. Even if maternal indirect effect dominates paternal indirect effect, our approach still provides an unbiased estimate for the variance of average indirect effect.

One implication of having sibling pairs instead of independent samples or trios in the analysis is that we also need to consider the shared environmental effects between siblings. Error terms on two traits (i.e., *ϵ*_1_ and *ϵ*_2_) are both normally distributed random variables. But we allow 1) correlation of errors on two traits for the same individual, which is a common assumption in the genetic correlation estimation literature [5,6], and importantly, we also allow 2) correlation of error terms between siblings due to their shared environments. More specifically, we assume:

$$\epsilon_{k}∼N(0,I_{N}⊗\Sigma_{k}),where\;\Sigma_{1}=\left[\begin{array}{cc} \sigma_{\epsilon_{k}}^{2} & \rho_{\epsilon_{k}}\\ \rho_{\epsilon_{k}} & \sigma_{\epsilon_{k}}^{2} \end{array}\right],\;k=1,2$$

We denote *ϵ*_1*i*_, *ϵ*_2*i*_ as such family i sibling pair error terms for trait 1 and 2 respectively, assume $\left[\begin{array}{c} \epsilon_{1i}\\ \epsilon_{2i} \end{array}\right]∼N(0,\left[\begin{array}{cc} \Sigma_{1} & \Sigma_{12}\\ \Sigma_{12} & \Sigma_{2} \end{array}\right])$, where $\Sigma_{12}=\left[\begin{array}{cc} \eta & \delta \\ \delta & \eta \end{array}\right]$.

### Parameter estimation

For simplicity, we denote the whole set of 14 parameters as $\Theta =\{\sigma_{\alpha_{1}}^{2},\sigma_{\beta_{1}}^{2},\rho_{\epsilon_{1}},\rho_{\alpha },\rho_{\beta },\eta ,\delta ,\sigma_{\alpha_{2}}^{2},\sigma_{\beta_{2}}^{2},\rho_{\epsilon_{2}},\rho_{\alpha_{1}\beta_{2}},\rho_{\alpha_{1}\beta_{1}},\rho_{\alpha_{2}\beta_{2}},\rho_{\alpha_{2}\beta_{1}}\}$, and use *Θ*_*s*_ to denote the kth parameter. Rather than utilizing an iterative restricted maximum likelihood algorithm to solve the linear mixed model, we employ the method of moments to improve computational efficiency. This approach produces parameter estimates by minimizing the distance between the model-derived variance-covariance matrix and cross-product matrix of phenotypes, i.e., *cov*(*Y*) where $Y={\left[Y_{1}^{T},Y_{2}^{T}\right]}^{T}$. Since we assumed an additive penetrance model, we can show that the model-derived covariance also follows an additive form:

$$cov(Y)=I+\sum_{s}\tilde{V_{s}}\Theta_{s}$$

where $\tilde{V_{s}}$ denote the sample relatedness matrix corresponding to s-th parameter. For example, $\tilde{V_{1}}=\frac{1}{M}\left[\begin{array}{cc} XX^{T} & 0\\ 0 & 0 \end{array}\right]-I_{4N}$ and $\tilde{V_{2}}=\frac{1}{M}\left[\begin{array}{cc} (X_{m}+X_{p}){\left(X_{m}+X_{p}\right)}^{T} & 0\\ 0 & 0 \end{array}\right]-I_{4N}$. We list all the relatedness matrices in **S4 Table**. Notice that the variances of error terms are not included in the parameter list. We replace them by one minus the rest of the variance terms since the phenotypic variance is standardized to be one. The cross-product matrix of phenotypes is the estimate of *cov*(*Y*) based on real data, i.e., $\hat{cov}(y)=yy^{T}$. We estimate all parameters by minimizing the following function

$$L(\Theta )=||yy^{T}-I-\sum_{s}\Theta_{s}\tilde{V_{s}}|{|}_{F}^{2}$$

where ‖∙‖_*F*_ is the Frobenius norm. To minimize this function, we use the gradient descent method. That is, we let $\frac{\partial L}{\partial \Theta_{s}}=0$ for all k and obtain a linear system of the form A*Θ* = *B*, where A is a matrix and B is a vector. Then we could obtain parameter estimates by solving this linear equation.

While the variance of each parameter estimate can be derived under this statistical framework, they can be numerically unstable (and even turn negative) when sample size is limited. Therefore, we applied a resampling-based block Jackknife approach [48] to quantify the variance of parameter estimates. Block Jackknife generates variance estimates by dividing the whole dataset into B blocks, holding out one block and producing parameter estimates using the other B-1 blocks in each time, then repeating this procedure for B times. Finally, we estimate variances using following formula:

$$\hat{var}({\hat{\Theta }}_{s})=\frac{B-1}{B}\sum_{b=1}^{B}({\hat{\Theta }}_{s,b}-{\bar{\Theta }}_{k}{)}^{2}$$

where ${\hat{\Theta }}_{s,b}$ denotes the estimate for s-th parameter in b-th repeat, and ${\bar{\Theta }}_{s}$ denotes the averaged value across B estimates for parameter *Θ*_*s*_. To account for multiple testing in real data applications, we calculated false discovery rate (FDR) based on all estimates for all pairs of traits.

Our model can also incorporate fixed effect covariates [49]. For simplicity, we ignore the subscripts in the proposed model and add fix effects as follows:

$$Y=Z\gamma +X\alpha +(X_{p}+X_{m})\beta +\epsilon$$

where *Z* denotes the covariate data matrix and *γ* denotes their fixed effects. To account for fixed effects, we can multiply a matrix *Q* that satisfies *QZ* = 0 to both sides of the equation. Matrix *Q* could be obtained by deriving the orthogonal vector spaces of the singular value decomposition of matrix *Z*. After multiplying matrix *Q* on both sides, the equation becomes the following without a fixed effect term.


$$QY=QX\alpha +Q(X_{p}+X_{m})\beta +Q\epsilon$$

Then, the estimation procedure for all parameters is the same as before.

### Reconstructing heritability and genetic covariance using decomposed variance components

Following the model described above, total heritability *h*^2^ and genetic covariance *ρ*_*gc*_ can be recovered from the decomposed variance component parameters as follows:

$$h_{i}^{2}=\sigma_{\alpha_{i}}^{2}+\sigma_{\beta_{i}}^{2}+2\rho_{\alpha_{i}\beta_{i}},\;i=1,2$$

$$\rho_{gc}=\rho_{\alpha }+\rho_{\beta }+\rho_{\alpha_{1}\beta_{2}}+\rho_{\alpha_{2}\beta_{1}}$$

By plugging in empirical estimates on the right-hand side of these equations, we could obtain estimates for total heritability and genetic covariance (**Fig 2**).

### Data processing

UKB samples with European ancestry were identified from principal component analysis (data field 22006). We used KING [50] to infer the pairwise family kinship and created family identifiers in UKB. Sum of parental genotypes were imputed by SNIPar [36]. Only autosomal SNPs with minor allele frequencies (MAF) greater than or equal to 0.05 and missing rate less than 0.01 were used for the analysis. Genotypes were standardized to have mean 0 and variance 1 using estimated minor allele frequencies, and missing values were imputed as 0. The sum of parental genotypes was standardized to have a variance of 2.

For phenotypes, participants were selected as overlapping samples of five phenotypes: height, BMI, EA, income, and overall health. We obtained height and BMI phenotypes from UKB data fields 12144 and 21001. Following previous work [51], we used data field 6138 to compute the EA phenotype as years of schooling. Household income data were obtained from UKB data field 738. The answers were coded as follows: (1)–Less than 18,000 (Pounds), (2)– 18,000 to 30,999, (3)– 31,000 to 51,999, (4)– 52,000 to 100,000, (5)–Greater than 100,000. Overall health was defined based on data field 100508. The answers were coded as follows: (1)–Excellent, (2)–Good, (3)–Fair, (4)–Poor. Note that a higher value indicates poorer health. We removed individuals with missing phenotype values and standardized all 5 traits to have mean 0 and variance 1. The final dataset we used for the analysis includes 4,748,473 SNPs and 12,571 sibling pairs (25,142 individuals and their imputed parents).

### Simulation settings

We utilized imputed genotypes in UKB as input. SNPs with missing rate greater than 5% and minor allele frequency less than 5% were removed from the dataset. 2000 full sibling pairs were randomly selected in UKB. We simulated phenotypes using real genotypes and preset parameters. A total of four settings (i.e. four sets of preset parameter values) were explored. The parameter values for each setting are listed in **S1 Table**. All settings were repeated 200 times. In each repeat, we simulated phenotypes by generating indirect effects, direct effects, and error terms based on the proposed penetrance model using the true parameters. Then, we produced parameter estimates using our statistical framework, and obtained variance estimates using block Jackknife.

## Supporting information

S1 Fig Parameter estimates in simulations. Panels A and B show results for simulation settings 1 and 2, respectively. Red dots denote pre-determined true parameter values in simulations.
(TIF)

S1 Table Parameter settings for simulations. (XLSX)

S2 Table Parameter estimates for single traits analysis. (XLSX)

S3 Table Parameter estimates for trait pair analysis. (XLSX)

S4 Table Sample relatedness matrices for cov(Y). (PDF)
