# Supplementary figures and images for: Decomposing heritability and genetic covariance by direct and indirect effect paths
Source: PLoS Genet. 2023 Jan 23;19(1):e1010620. doi: 10.1371/journal.pgen.1010620 (PMC9894552; doi:10.1371/journal.pgen.1010620)

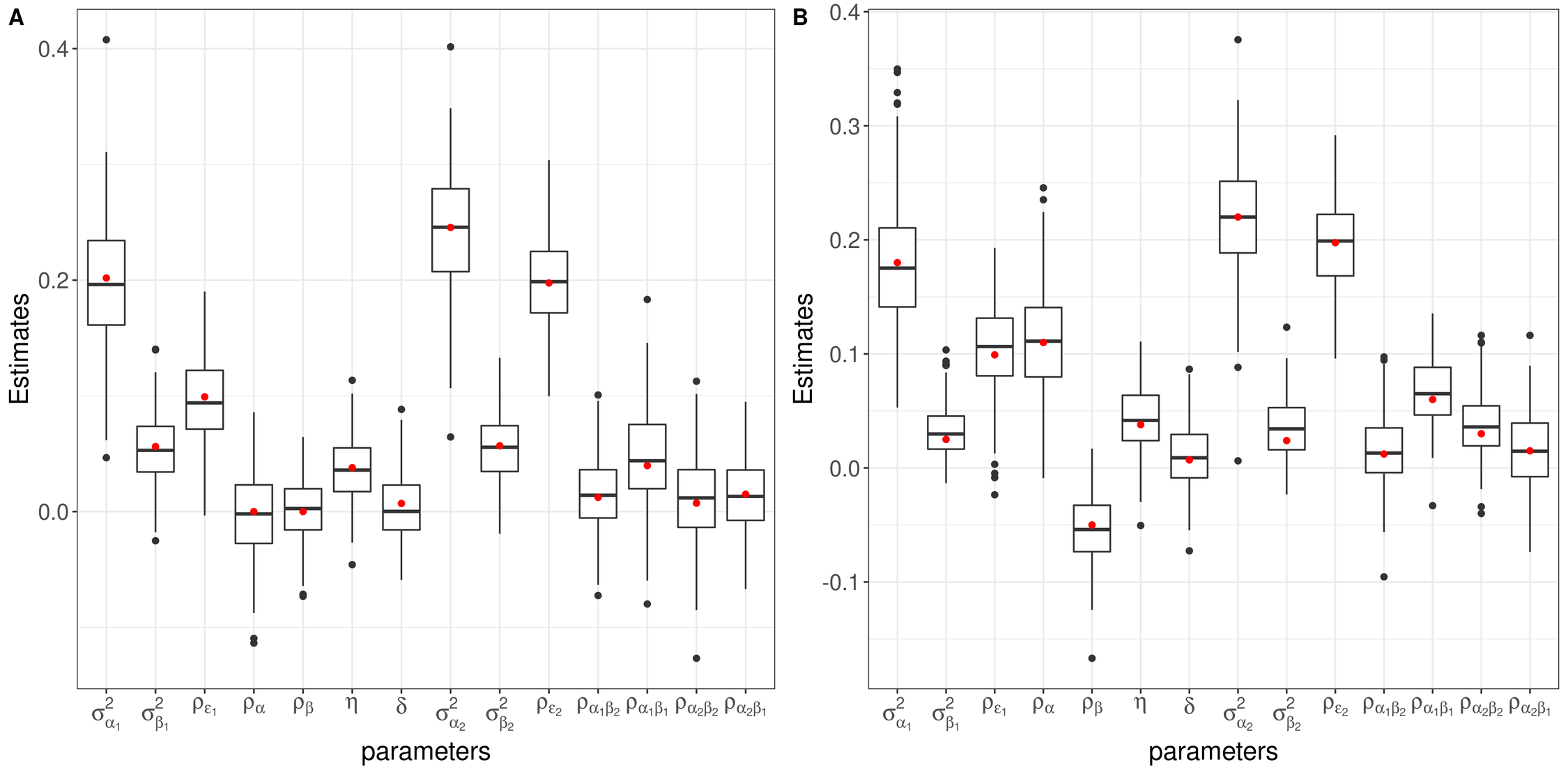

Supplement: S1 Fig — Panels A and B show results for simulation settings 1 and 2, respectively. Red dots denote pre-determined true parameter values in simulations. (TIF) [file pgen.1010620.s001.tif]
