# Supplementary material for: Decomposing heritability and genetic covariance by direct and indirect effect paths
Source: PLoS Genet. 2023 Jan 23;19(1):e1010620. doi: 10.1371/journal.pgen.1010620 (PMC9894552; doi:10.1371/journal.pgen.1010620)
Supplement: S4 Table — (PDF) [file pgen.1010620.s005.pdf]

Table S4. Sample relatedness matrices for cov(Y).

NOTATION	ESTIMATED PARAMETERS	RELATEDNESS MATRIX
$\widetilde{V_1}$	variance of direct effect on trait 1	$\frac{1}{M}\begin{bmatrix} XX^T & 0 \\ 0 & 0 \end{bmatrix} - I_{4N}$
$\widetilde{V_2}$	variance of indirect effect on trait 1	$\frac{1}{M}\begin{bmatrix} (X_m+X_p)(X_m+X_p)^T & 0 \\ 0 & 0 \end{bmatrix} - 2I_{4N}$
$\widetilde{V_3}$	covariance of error terms for trait 1	$\begin{bmatrix} I_N \otimes \begin{bmatrix} 0 & 1 \\ 1 & 0 \end{bmatrix} & 0 \\ 0 & 0 \end{bmatrix}$
$\widetilde{V_4}$	covariance of direct effects between two traits	$\frac{1}{M}\begin{bmatrix} 0 & XX^T \\ XX^T & 0 \end{bmatrix}$
$\widetilde{V_5}$	covariance of indirect effects between two traits	$\frac{1}{M}\begin{bmatrix} 0 & (X_m+X_p)(X_m+X_p)^T \\ (X_m+X_p)(X_m+X_p)^T & 0 \end{bmatrix}$
$\widetilde{V_6}$	covariance of error terms for the same individual in two traits	$\begin{bmatrix} 0 & I_{2N} \\ I_{2N} & 0 \end{bmatrix}$
$\widetilde{V_7}$	covariance of error terms between two siblings in two traits	$\begin{bmatrix} 0 & I_N \otimes \begin{bmatrix} 0 & 1 \\ 1 & 0 \end{bmatrix} \\ I_N \otimes \begin{bmatrix} 0 & 1 \\ 1 & 0 \end{bmatrix} & 0 \end{bmatrix}$
$\widetilde{V_8}$	variance of direct effect on trait 2	$\frac{1}{M}\begin{bmatrix} 0 & 0 \\ 0 & XX^T \end{bmatrix} - I_{4N}$
$\widetilde{V_9}$	variance of indirect effect on trait 2	$\frac{1}{M}\begin{bmatrix} 0 & 0 \\ 0 & (X_m+X_p)(X_m+X_p)^T \end{bmatrix} - 2I_{4N}$
$\widetilde{V_{10}}$	covariance of error terms for trait 2	$\begin{bmatrix} 0 & 0 \\ 0 & I_N \otimes \begin{bmatrix} 0 & 1 \\ 1 & 0 \end{bmatrix} \end{bmatrix}$
$\widetilde{V_{11}}$	covariance of direct effect of trait 1 and indirect effect of trait 2	$\frac{1}{M}\begin{bmatrix} 0 & X(X_m+X_p)^T \\ (X_m+X_p)X^T & 0 \end{bmatrix}$
$\widetilde{V_{12}}$	covariance of direct effect of trait 1 and indirect effect of trait 1	$\frac{1}{M}\begin{bmatrix} (X_m+X_p)X^T + X(X_m+X_p)^T & 0 \\ 0 & 0 \end{bmatrix} - 2I_{4N}$
$\widetilde{V_{13}}$	covariance of direct effect of trait 2 and indirect effect of trait 2	$\frac{1}{M}\begin{bmatrix} 0 & 0 \\ 0 & (X_m+X_p)X^T + X(X_m+X_p)^T \end{bmatrix} - 2I_{4N}$
$\widetilde{V_{14}}$	covariance of direct effect of trait 2 and indirect effect of trait 1	$\frac{1}{M}\begin{bmatrix} 0 & (X_m+X_p)X^T \\ X(X_m+X_p)^T & 0 \end{bmatrix}$
